# Comparative Transcriptomic Analysis of Race 1 and Race 4 of *Fusarium oxysporum* f. sp. *cubense* Induced with Different Carbon Sources

**DOI:** 10.1534/g3.117.042226

**Published:** 2017-05-03

**Authors:** Shiwen Qin, Chunyan Ji, Yunfeng Li, Zhenzhong Wang

**Affiliations:** * Laboratory of Physiological Plant Pathology, South China Agricultural University, Guangzhou 510642, China; † Guangdong Province Key Laboratory of Microbial Signals and Disease Control, South China Agricultural University, Guangzhou 510642, China

**Keywords:** *Fusarium oxysporum* f. sp. *cubense*, transcriptome, carbohydrate-active enzymes, cell wall-degrading enzymes, pathogenicity genes

## Abstract

The fungal pathogen *Fusarium oxysporum* f. sp. *cubense* causes *Fusarium* wilt, one of the most destructive diseases in banana and plantain cultivars. Pathogenic race 1 attacks the “Gros Michel” banana cultivar, and race 4 is pathogenic to the Cavendish banana cultivar and those cultivars that are susceptible to Foc1. To understand the divergence in gene expression modules between the two races during degradation of the host cell wall, we performed RNA sequencing to compare the genome-wide transcriptional profiles of the two races grown in media containing banana cell wall, pectin, or glucose as the sole carbon source. Overall, the gene expression profiles of Foc1 and Foc4 in response to host cell wall or pectin appeared remarkably different. When grown with host cell wall, a much larger number of genes showed altered levels of expression in Foc4 in comparison with Foc1, including genes encoding carbohydrate-active enzymes (CAZymes) and other virulence-related genes. Additionally, the levels of gene expression were higher in Foc4 than in Foc1 when grown with host cell wall or pectin. Furthermore, a great majority of genes were differentially expressed in a variety-specific manner when induced by host cell wall or pectin. More specific CAZymes and other pathogenesis-related genes were expressed in Foc4 than in Foc1 when grown with host cell wall. The first transcriptome profiles obtained for Foc during degradation of the host cell wall may provide new insights into the mechanism of banana cell wall polysaccharide decomposition and the genetic basis of Foc host specificity.

The soil-borne fungus *Fusarium oxysporum* f. sp. *cubense* (Foc) causes *Fusarium* wilt, one of the most important lethal diseases in banana, which has a devastating effect on banana production worldwide (Ploetz 2006, 2015a). According to the susceptibility of specific banana cultivars, Foc can be divided into four races (Stover and Buddenhagen 1986; Stover 1990). Race 1 (Foc1) causes disease in the “Gros Michel” (AAA) and “Silk” (AAB) cultivars (Waite and Stover 1960). Race 2 is the causal agent of this disease in the hybrid triploid Bluggoe (ABB). Race 3 is virulent only to *Heliconia* spp. and does not affect *Musa* spp. (Stover 1962). Race 4 (Foc4) infects Cavendish (AAA) cultivars and those cultivars susceptible to races 1 and 2 (Su *et al.* 1986). In the middle of the past century, Foc1 devastated the Gros Michel cultivar, which was the main commercial banana cultivar grown for the export trade (Stover 1962). This forced the trade to shift to resistant cultivars of the Cavendish variety (Buddenhagen 1990). However, the virulent strain Foc4, to which Cavendish is susceptible, spread rapidly to banana-growing regions and now threatens the global production of Cavendish and other currently popular cultivars (Ploetz 2006; Butler 2013). Regardless of biological, chemical, or cultural measures, no appropriate management strategies for *Fusarium* wilt are currently available to eliminate this pathogen once plants are infected (Ploetz 2007, 2015b).

Despite the importance and necessity of controlling this disease, the molecular mechanisms of pathogenesis in banana and the genetic basis for host specificity are still poorly understood. Only a few pathogenicity genes involved in Foc4 infection of Cavendish cultivars have been identified thus far, which include the following: *Foatf1*, encoding a basic leucine zipper (bZIP) transcription factor (TF); *FoOCH1*, encoding a putative α-1,6-mannosyltransferase; *FoSlt2*, *FoMkk2*, and *FoBck1*, encoding mitogen-activated protein kinases (MAPKs); and four G-protein subunit genes, *FGA1*, *FGA2*, *FGA3*, and *FGB1* (Qi *et al.* 2013; Li *et al.* 2014; Ding *et al.* 2015; Guo *et al.* 2016a,b).

Recently, with the extensive application of next-generation sequencing technologies to plant phytopathogen genomics, increasing numbers of gene sets have been found to be involved in pathogenicity and pathogen–plant interactions with essential roles in plant disease. The genomes of *F. oxysporum* isolates, including *F. oxysporum* f. sp. *lycopersici* (Fol), *F. oxysporum* f. sp. *cubense*, *F. oxysporum* f. sp. *ciceris*, and *F. oxysporum* f. sp. *melonis*, have been sequenced and have been shown to contain a large number of pathogenicity genes and virulence-related genes, including predicted effectors, TFs, transmembrane transporters, and CAZymes, among others (Ma *et al.* 2010; Guo *et al.* 2014; van Dam *et al.* 2016; Williams *et al.* 2016). A significant finding is that lineage-specific genes on mobile and partly dispensable chromosomes can be transferred intraspecifically and possibly interspecifically, thus constituting determinants of pathogenicity and host range, which have been identified in *Fusarium* species and other fungal species (Han *et al.* 2001; Chuma *et al.* 2003; Coleman *et al.* 2009; Ma *et al.* 2010; Schmidt *et al.* 2016; Williams *et al.* 2016). Whole-genome sequence analysis of Foc1 and Foc4 has led to the identification of a large number of potential pathogenicity genes. Although the genomes of the two Foc races are highly syntenic with Fol, neither contains the lineage-specific genomic regions that are exclusive to Fol (Guo *et al.* 2014).

Transcriptomic analysis of phytopathogens is also one of the most effective ways to obtain a full understanding of fungal pathogenesis at the molecular level, potentially allowing the characterization of all actively expressed genes and transcripts at different stages of plant infection or under various conditions. In *Fusarium* species, transcriptomic studies have mainly focused on the facultative pathogen *F. graminearum* under a variety of culture conditions (different carbon and nitrogen sources) *in vitro* and during different stages of the infection of host plants *in planta* (Guldener *et al.* 2006; Seong *et al.* 2008; Gardiner *et al.* 2009; Lysoe *et al.* 2011; Carapito *et al.* 2013; Boedi *et al.* 2016). Based on these studies, specific subsets of *F. graminearum* genes are considered to be pathogenicity-related or virulence genes. However, there are few transcriptomic resources available for Foc in the NCBI database. To date, only the transcriptome of Foc during early Cavendish variety infection has been examined. This study indicated that pathogenicity genes encoding MAPK, G-proteins, and a two-component system involved in signaling are activated in Foc4 rather than Foc1 during early Cavendish variety infection (Guo *et al.* 2014). However, more information about the changes in the expression of pathogenicity-related genes at different critical points in disease establishment and under various growth conditions *in vitro*, as well as the molecular basis of the difference in virulence between the two races, is still needed.

The plant cell wall is primarily composed of three types of polysaccharides: cellulose, hemicellulose, and pectin. It is the first physical and chemical barrier against pathogen invasion but is also a nutrient source for invading pathogens. Pectin, the most sophisticated polysaccharide in terms of structure, is a highly diverse polymer that contains several specific polysaccharides (Pérez *et al.* 2000; Caffall and Mohnen 2009). Many pathogenic fungi have the genetic potential to decompose plant cell wall polysaccharides by producing an extensive set of CAZymes (Aro *et al.* 2005; Coutinho *et al.* 2009; van den Brink and de Vries 2011). The plant cell wall-degrading enzymes (CWDEs) secreted by fungi are all assigned to CAZymes, which are one type of pathogenicity factor used by pathogens or saprophytes to invade plants (Götesson *et al.* 2002; Ospina-Giraldo *et al.* 2003, 2009). The genomes of Foc1 and Foc4 contain large proportions of CAZymes, implying that effective banana cell wall degradation may be important for the virulence of Foc (Guo *et al.* 2014).

In the current study, to go a step further in the analysis of Foc genes that participate in host cell wall decomposition and contribute to pathogenicity in the banana host, RNA-seq was used to compare the global transcriptional profiles of Foc1 and Foc4 cultured in media containing banana cell wall, pectate, or glucose as the sole carbon source. The aims were as follows: (1) to elucidate the mechanism of the Foc genes involved in the decomposition of different polysaccharides from the primary plant cell wall; (2) to identify differences in gene contents and transcriptional levels between the two Foc races that might account for the variations in the virulence of Foc races during infection of their particular hosts; and (3) to identify Foc pathogenicity genes responsible for decomposition of the host cell wall. This work will provide further transcriptomic information on the mechanisms of *Fusarium*–plant interactions and contribute to the development of feasible strategies for controlling Foc infection.

## Materials and Methods

### Fungal strains and growth conditions

The “Brazil” banana cultivar (AAA group, Cavendish) is Foc4-susceptible, but highly resistant to Foc1, while the “Fenjiao” cultivar (ABB group, Pisang Awak) is susceptible to both Foc4 and Foc1. Single-conidium pure cultures of the Foc1 C2 and Foc4 DZ1 strains were isolated from diseased rhizome tissues of the Fenjiao and Brazil cultivars, respectively. Both Foc isolates underwent molecular characterization of Foc and were maintained as microconidial suspensions in 30% (v/v) glycerol at −80° in our laboratory. Foc isolates were periodically inoculated onto their respective host banana cultivars in a growth chamber to confirm their pathogenicity. The Foc4 DZ1 strain was identified as *F. oxysporum* f. sp. *cubense* tropical race 4 (Foc TR4). Banana cell walls of the Brazil and Fenjiao cultivars were prepared from greenhouse-grown 6-wk-old banana plants using the method described for maize (Sposato *et al.* 1995).

Foc isolates (10^6^ conidia) were shaken in synthetic medium (SM) (Garcia Maceira *et al.* 1997) supplemented with 1% [w/v] glucose at 28° at 120 rpm. After 48 hr of growth, the mycelium was harvested, washed three times in sterile water, and transferred to fresh SM supplemented with different carbon sources: 1% [w/v] glucose, 1% [w/v] citrus pectin (Sigma), or 1% banana cell wall (cell wall from the Brazil banana cultivar for Foc4 or from Fenjiao for Foc1). The mycelia were shaken at 28° at 120 rpm for 24 hr and were then collected using previously sterilized tweezers, washed three times with sterile water treated with diethylpyrocarbonate (DEPC, Sigma), filtered through nitrocellulose membranes (0.22 μm), and flash frozen in liquid nitrogen.

### RNA extraction, library preparation, and sequencing of the Foc transcriptome

Total RNA from three independent cultures grown under each condition was extracted separately using the TRIzol Reagent (Invitrogen) following the manufacturer’s instructions, then treated with RNase-free DNase I (TaKaRa, China). One part of total RNA from three independent cultures for each condition were pooled in equal amounts for RNA-seq analysis, and the other part was stored at −80° for further real-time quantitative PCR (qRT-PCR) verification. The quality and quantity of the total RNA was validated using agarose gel electrophoresis and a 2100 Bioanalyzer (Agilent Technologies). A total of 3 μg high-quality RNA (RNA integrity number > 8) per sample was prepared to generate cDNA libraries. Sequencing libraries were generated using the NEBNext Ultra RNA library prep kit for Illumina (New England BioLabs). The cDNA libraries were then sequenced on the Illumina HiSequation 2000 platform using a 100 bp paired-end sequencing strategy at the Novogene Corporation, Beijing, China.

### Transcriptome assembly and analysis

Clean reads were obtained for the six libraries by removing reads containing adapters, poly-Ns, and low-quality bases from the raw reads. The sequencing quality of the clean reads was calculated at the Q20, Q30, GC-content, and sequence duplication levels. *De novo* transcriptome assembly was performed with Trinity (Grabherr *et al.* 2011). After sequence assembly, unigenes with the expected length and size were generated from the longest transcripts at each locus.

All unigenes in the Foc transcriptome were aligned to protein databases with a priority order of the NCBI nonredundant protein (Nr) database, Swiss-Prot, Kyoto Encyclopedia of Genes and Genomes (KEGG), and the EuKaryotic Orthologous Groups (KOG) database via BLASTX alignment (*E*-value ≤ 1 × 10^−5^) (Altschul *et al.* 1997). The coding sequences (CDSs) and corresponding amino acid sequences of the unigenes were extracted from the BLAST analysis results. For the unigenes with no hits in BLAST, ESTScan software was used to predict their CDSs and corresponding amino acid sequences (Iseli *et al.* 1999). The protein families of all unigenes were annotated through Pfam analysis (Finn *et al.* 2014). Gene ontology (GO) annotations according to the biological process, cellular component, and molecular function ontologies were assigned for each unigene using the Blast2GO program (Conesa *et al.* 2005). The nucleotide sequences of all unigenes were aligned to the NCBI nucleotide sequences (Nt) database with the BLAST algorithm (*E*-value ≤ 1 × 10^−5^). CAZymes were identified and classified using the CAZymes Analysis Toolkit (Park *et al.* 2010), applying a cut-off *E*-value ≤ 1 × 10^−5^, and through CAZyme annotation (dbCAN) (Yin *et al.* 2012). Foc pathogenicity genes were predicted using Blastp (*E*-value ≤ 1 × 10^−5^), against protein sequences in the pathogen–host interaction database (Winnenburg *et al.* 2006).

### Gene expression profiling analyses

To estimate the RNA transcriptional abundance for each gene, the clean reads from six Foc libraries were mapped back to the reference-assembled transcriptome using RSEM (Li and Dewey 2011). Gene expression levels were calculated as FPKM values (expected fragments per kilobase of transcript per million fragments sequenced) using the program Cufflinks (Trapnell *et al.* 2010). The read counts from six libraries were first adjusted using the edgeR program package through one scaling-normalized factor (Robinson *et al.* 2010). Then, the differential expression levels of each unigene were analyzed using the DESeq R package by employing a negative binomial distributions model (Anders and Huber 2010; Wang *et al.* 2010). Differentially expressed genes (DEGs) were identified using a *q*-value (an adjusted p-value) ≤ 0.005 and a |log_2_(fold change)| ≥ 1 as the threshold (Storey and Tibshirani 2003). To further characterize the biological functions and metabolic pathways of DEGs, GO enrichment analyses were performed using Goseq (Young *et al.* 2010), and KEGG pathway enrichment analysis was carried out using KOBAS (Mao *et al.* 2005), with a cut-off p-value ≤ 0.05.

### qRT-PCR verification

For validation of the RNA-seq results, 15 genes with different expression levels and diverse functions were randomly selected for qRT-PCR analysis. The total RNA samples (pretreated with DNase I) were the same samples obtained from three independent cultures for each condition but were not pooled, as for RNA-seq. Total RNA was reverse transcribed in a 20 μl reaction mixture using the PrimeScript RT Master Mix Kit (TaKaRa, China). The qRT-PCR was performed using a Thermal Cycler Dice TP900 (Takara, Japan) in combination with the SYBR Premix Ex Taq II Kit (TaKaRa, China) according to the manufacturer’s protocol. The gene-specific primers designed for qRT-PCR analysis are listed in Supplemental Material, Table S10 in File S2. The qRT-PCR conditions were as follows: initial denaturation at 95° for 30 sec, followed by 40 cycles of 95° for 5 sec, and 60° for 30 sec. All amplification reactions were run in triplicate with three biological replicates. The expression levels of target genes in response to growth in the presence of host cell wall or pectin were normalized to the constitutively expressed β-tubulin gene (reference gene), and were calibrated compared with the levels recorded during growth in glucose culture (set as 1) using the 2^−ΔΔ^*^Ct^* method (Livak and Schmittgen 2001).

### Data availability

Strains of Foc used in this study are available upon request. The raw RNA-seq reads from the six samples are available at the NCBI Sequence Read Archive database under the accessions SRA486974. Supplemental figures and supplemental tables contain detailed information of all supplemental files to support this study.

## Results

### RNA sequencing and de novo assembly of the Foc transcriptome

To obtain a global view of the Foc1 and Foc4 transcriptomes during host cell wall degradation, RNA-seq was performed on Foc1 and Foc4 cultures containing banana cell wall, pectin, and glucose as the sole carbon source, and the gene expression profiles of each strain were compared. Six cDNA libraries prepared from the pooled total RNA extracted from two Foc isolates grown under three carbon conditions were paired-end sequenced on the Illumina HiSequation 2000 platform. The sequencing results for the six libraries (designated G_Foc1, G_Foc4, P_Foc1, P_Foc4, FCW_Foc1, and BCW_Foc4) are summarized in Table 1. After filtering out repetitive, low-complexity, and low-quality reads, the clean reads were assembled into 38,950 transcripts with an average length of 1937 bp and an N50 length of 3235 bp. The longest transcript for each locus was defined as the unigene, resulting in 23,417 unigenes with an average length of 1419 bp and an N50 length of 2542 bp (Table 2). The size distribution of these transcripts and unigenes is shown in Figure S1 in File S1.

**Table 1:** The six Foc libraries quality of RNA-seq

Sample*^a^*	Raw Reads	Clean Reads	Clean Bases	Error (%)	Q20 (%)	Q30 (%)	GC Content (%)
G_Foc1	75163928	70921444	7.10G	0.035	97.39	91.33	52.35
G_Foc4	67448538	62516786	6.26G	0.035	97.39	91.33	52.55
P_Foc1	63999398	60148382	6.02G	0.035	97.37	91.28	52.79
P_Foc4	69432346	65433836	6.54G	0.035	97.36	91.22	52.66
FCW_Foc1	79810356	75082316	7.50G	0.040	97.30	91.05	53.24
BCW_Foc4	58060848	55168204	5.52G	0.040	97.33	91.19	53.21

a G, glucose; P, pectin; FCW, cell wall of Fengjiao cultivar; BCW, cell wall of Brazil cultivar.

**Table 2:** Length distribution of assembled transcripts and unigenes

	Transcript	Unigene
Total number of transcripts or unigenes	38,950	23,417
Number of transcripts or unigenes (200–500 bp)	10,082	8,810
Number of transcripts or unigenes (500–1000 bp)	5,945	3,971
Number of transcripts or unigenes (1000–2000 bp)	9,003	5,045
Number of transcripts or unigenes (> 2000 kbp)	13,920	5,591
Total length (bp)	75,459,645	33,233,628
Min length (bp)	201	201
Max length (bp)	24,163	24,163
Mean length (bp)	1,937	1,419
N50 (bp)*^a^*	3,235	2,542
N90 (bp)*^a^*	1,010	605

a The N50 size is computed by sorting all transcripts from largest to smallest and by determining the minimum set of transcripts whose sizes total 50% of the entire transcript and unigene was the same; N90 was counted in a similar way.

### Functional gene annotation

The functional annotation of all 23,417 assembled unigenes was mainly based on BLAST homology searches against seven public protein databases. A total of 20,879 unigenes were annotated in at least one database, accounting for 89.16% of the unigenes (Table S1 in File S2). Among these unigenes, 15,883 (67.82%) showed high homology with known proteins in the Nr database, and 8212 unigenes (35.06%) were annotated in the Swiss-Prot database.

**Figure 1:**
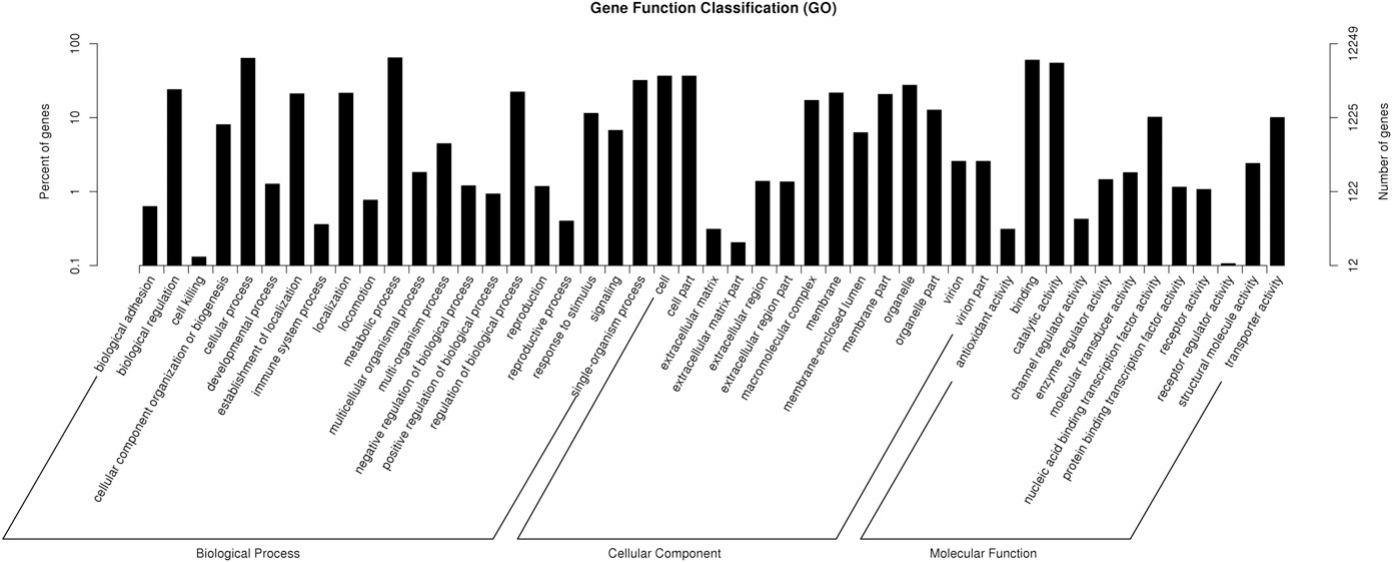
Gene Ontology (GO) categories assigned to the Foc unigenes. The right *y*-axis indicates the number of genes in a category. The left *y*-axis indicates the percentage of a specific category of genes in that main category.

GO assignments were utilized to predict the functions of Foc unigenes by classifying them according to biological processes. A total of 12,249 unigenes (52.3%) were assigned to at least one GO term and were categorized into 54 functional groups in three categories (biological process, cellular component, and molecular function) (Figure 1 and Table S2 in File S2). Under biological processes, metabolic process and cellular process were the most prominent GO terms. Within the molecular function ontology, genes associated with the “binding,” “catalytic,” “nucleic acid binding transcription factor activity,” and “transporter activity” groups were the most abundant. These results indicate that Foc grows rapidly and exhibits extensive metabolic activity under each of the three tested carbon source conditions.

Using the KOG database to further annotation, a total of 5411 unigenes (23.1%) were clustered into 25 KOG categories (Figure S2 in File S1). Among these categories, the majority of genes were associated with “general functional prediction only” (1142 unigenes, 21.1%), “post-translational modification, protein turnover, and chaperones” (479 unigenes, 8.9%), and “secondary metabolite biosynthesis, transport, and catabolism” (458 unigenes, 8.5%).

**Figure 2:**
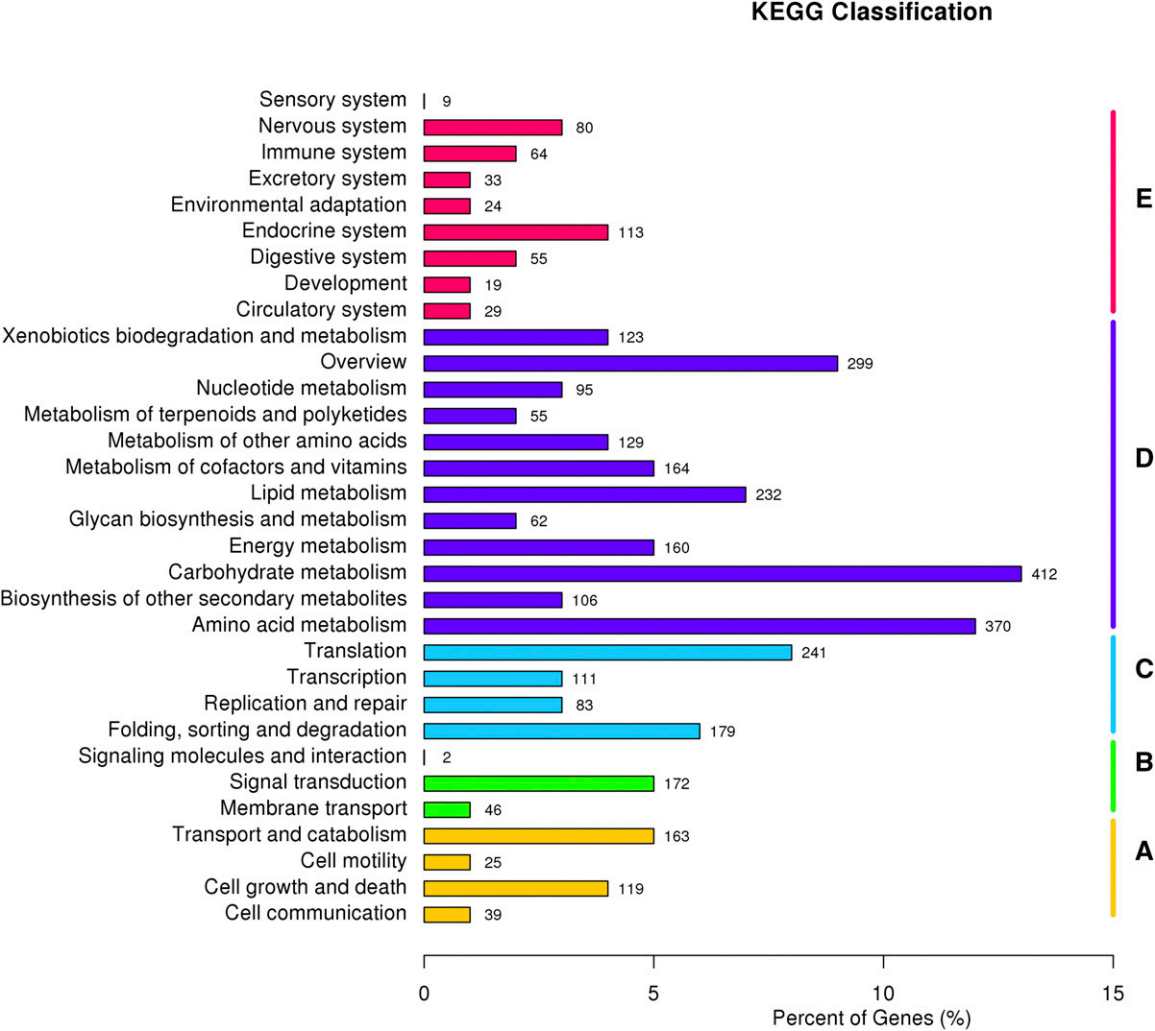
Pathways assignment based on the Kyoto Encyclopedia of Genes and Genomes (KEGG). A = Cellular Processes; B = Environmental Information Processing; C = Genetic Information Processing; D = Metabolism; and E = Organismal Systems.

To determine the biological functions and interactions of Foc unigenes in the three carbon source cultures, a total of 3194 unigenes were assigned to the 259 KEGG biochemical pathways (Figure 2). Most of the unigenes were related to the category “metabolism,” with pathways “carbohydrate metabolism” (412 unigenes) and “amino acid metabolism” (370 unigenes) being the most representative (Figure 2). KEGG pathways belonging to “carbohydrate metabolism,” such as “starch and sucrose metabolism” and “pentose and glucuronate interconversions,” were more likely to be involved in banana cell wall polysaccharide degradation by Foc (Table S3 in File S2).

### CAZymes present in the Foc transcriptome

CAZymes secreted by fungal pathogens can break down plant cell walls; therefore, they are important in establishing infection and in accessing nutrients during growth. CAZymes are assigned to CAZy families of glycoside hydrolases (GHs), carbohydrate esterases (CEs), polysaccharide lyases (PLs), glycosyl transferases (GTs), auxiliary activities (AAs), and carbohydrate-binding modules (CBMs). In this study, a total of 2105 CAZyme modules were identified among 1970 unigenes using the CAZymes Analysis Toolkit and dbCAN (Tables S4 and S5 in File S2). These unigenes were sorted into superfamilies, with GH being the most abundant, followed by GT, CE, CBM, AA, and PL (Table S4 in File S2). Based on the CAZyme modules for plant cell wall degradation, a total of 393 unigenes were identified as putative CWDEs, including 28 GHs, 3 CEs, 3 CBMs, 4 PLs, and 1 AA family (Table S6 in File S2). According to the substrates degraded by CWDEs, these putative CWDEs were categorized into 33 classes of enzymes (Table S6 in File S2). Unigenes encoding endo- and exopolygalacturonase were the most abundant, followed by β-1,4-xylosidase and β-1,4-glucosidase. These results indicate that Foc produces a large arsenal of plant CWDEs during degradation of host cell wall polysaccharides, with pectinase being the most abundant, followed by hemicellulase and cellulase.

### Pathogenicity-associated genes in the Foc transcriptome

The PHI database contains expert information on experimentally verified pathogenicity, virulence, lethal, and effector genes from fungal and oomycete pathogens that infect animal, plant, and fungal hosts. The annotation results showed that 1202 unigenes were characterized as known genes proven to affect the outcome of pathogen–host interactions in various pathogenic fungi. In addition, 432 unigenes homologous to PHI genes associated with loss of pathogenicity (86), reduced virulence (340), and pathogenic effectors (6) were considered to be pathogenicity determinants for Foc (Table S7 in File S2). These unigenes encoded effectors, G-proteins and G-protein-coupled receptors, signaling protein, TFs, CWDEs, cutinases, transporters, cytochrome P450s, polyketide synthases, and chitin synthases, among others (Table S8 in File S2). GO analysis revealed that Foc genes homologous to PHI genes fell into a variety of metabolic processes, thereby highlighting the important roles of these processes in pathogenicity (Figure S3 in File S1).

### DEGs induced by host cell wall or pectin and qRT-PCR verification

Having generated a Foc reference transcriptome, our next goal was to identify genes displaying significant changes in expression when Foc was grown with host cell wall polysaccharides. For each library, all clean reads were mapped back to the Foc reference transcriptome. The percentage of clean reads from each library that could be uniquely mapped ranged from 90.67 to 91.75%, thus providing good coverage of the transcript profiles (Table S9 in File S2). Six Foc libraries showed similar FPKM density distributions (Figure S4 in File S1). To identify expression patterns of similarity across six Foc samples, the principal component analysis and Pearson correlation analysis of scaled expression profiles revealed six distinct clusters when Foc1 and Foc4 grew on cultures containing three different carbon sources, suggesting that the gene expression profiles induced by host cell wall were significantly different as compared to those induced by pectin or glucose (Figure S5 in File S1). The major cause for this result was that the host cell wall is composed of different types of polysaccharides, including cellulose, hemicellulose, and pectin, which could induce more genes expression in Foc than pectin or glucose. Besides, the gene expression profiles of Foc4 were also significantly different from those of Foc1 induced by the same carbon source.

DEGs were analyzed via pairwise comparisons of P_Foc4 *vs.* G_Foc4, P_Foc1 *vs.* G_Foc1, P_Foc4 *vs.* P_Foc1, FCW_Foc1 *vs.* G_Foc1, BCW_Foc4 *vs.* G_Foc4, and BCW_Foc4 *vs.* FCW_Foc1. To verify the RNA-seq results, 15 unigenes were randomly selected for qRT-PCR analysis. These genes, which were involved in carbohydrate metabolism, signaling, and transport, or were genes of unknown function, were either upregulated, downregulated, or unaffected in the two races. Seventy-nine (87.7%) of the 90 qRT-PCR results agreed with the changes in transcript levels detected through RNA-seq (*r* = 0.89, p-value ≤ 10^−5^) (Figure S6 in File S1 and Table S10 in File S2). Thus, the RNA-seq data were considered reliable for the identification of DEGs induced by host cell wall and pectin in this study.

**Figure 3:**
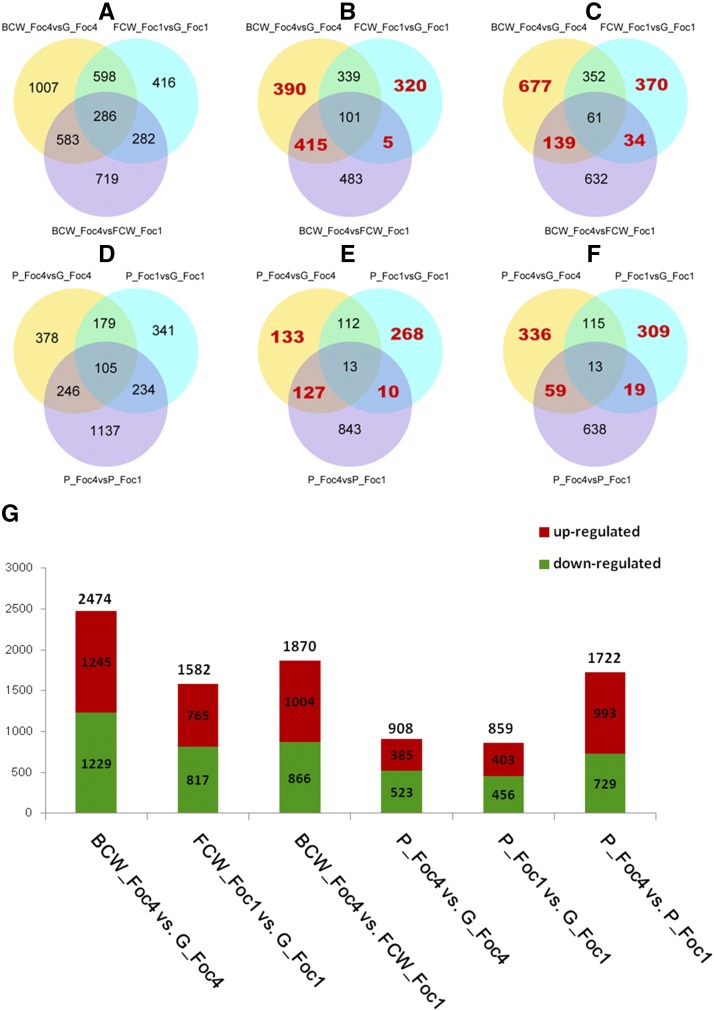
Number of differentially expressed genes (DEGs) in cells grown with host cell wall polysaccharides. The number of DEGs that were up- and downregulated in each comparison are shown in the Venn diagrams (A–F) and histogram (G). The number of DEGs (A), including upregulated (B) and downregulated DEGs (C), derived from comparing cells grown in the presence of host cell wall. The number of DEGs (D), including upregulated (E) and downregulated DEGs (F), derived from comparing cells grown in the presence of pectin. The numbers of specific DEGs regulated by Foc1 or Foc4 were shown in the nonoverlapping regions (red numbers). The DEGs were identified by applying a threshold of *q*-value ≤ 0.005 and an absolute value of |log_2_(fold-change)| ≥ 1.

The global gene expression profiles of each comparison are shown in Figure 3. Overall, a much larger number of genes showed altered expression levels in Foc4 (2474 DEGs) grown in the presence of host cell wall than in Foc1 (1582 DEGs). A slightly greater number of genes showed altered expression levels in Foc4 (908 DEGs) grown in the presence of pectin than in Foc1 (859 DEGs) (Figure 3). Additionally, the intensity of gene expression was different between the two races grown in the presence of host cell wall or pectin, which could be found by comparing BCW_Foc4 *vs.* FCW_Foc1 or P_Foc4 *vs.* P_Foc1. Compared with downregulated DEGs, more upregulated DEGs were found when comparing BCW_Foc4 *vs.* FCW_Foc1 or P_Foc4 *vs.* P_Foc1, which indicated that total mRNA expression in Foc4 was higher than in Foc1 (Figure 3). These results implied that Foc4 degrades host cell wall polysaccharides by changing the expression of more genes than Foc1. GO enrichment analyses showed that a large number of DEGs were significantly enriched in a variety of metabolic processes (Tables S11 and S12 in File S2). KEGG pathway enrichment analysis revealed that the specific metabolism pathways were significantly enriched among the DEGs regulated by Foc during degradation of host cell wall polysaccharides (Tables S13 and S14 in File S2).

### DEGs specifically found in Foc grown in the presence of host cell wall or pectin

The great majority of Foc genes are differentially expressed in a variety-specific manner during decomposition of the host cell wall. The numbers of specific DEGs up- and downregulated by Foc1 or Foc4 during the degradation of the host cell wall or pectin were shown in the nonoverlapping regions (red numbers) of the Venn diagram (Figure 3, B, C, E, and F and Table S15 in File S2). Compared with Foc1 grown with host cell wall, many more DEGs were specifically regulated in Foc4 (805 upregulated and 816 downregulated specific DEGs); more than twice as many DEGs were specifically regulated in Foc4 than in Foc1 (325 upregulated and 404 downregulated specific DEGs) (Figure 3, B and C and Table S15 in File S2). In contrast, the number of DEGs that were specifically regulated in Foc4 (260 upregulated and 395 downregulated specific DEGs) was greater than in Foc1 (278 upregulated and 328 downregulated specific DEGs) grown with pectin (Figure 3, D and F and Table S15 in File S2). Additionally, the gene expression levels of the specific DEGs in Foc4 were higher than in Foc1 (Figure 3, B, C, E, and F). More specific DEGs were upregulated in the comparison of BCW_Foc4 *vs.* FCW_Foc1 or P_Foc4 *vs.* P_Foc1.

Additionally, the annotation of specific DEGs showed that Foc4 specifically expresses more pathogenesis-related genes than Foc1 during degradation of host cell wall polysaccharides, which may account for the variation in the virulence of these races during infection of their particular hosts (Table 3). The number of specific DEGs related to fungal pathogenicity identified in the comparison of BCW_Foc4 *vs.* G_Foc4 (439 DEGs) was almost twice as great as in the comparison of FCW_Foc1 *vs.* G_Foc1 (219 DEGs) (Table 3). In particular, more specific DEGs annotated as G-protein, chitin synthase, ATP-binding cassette (ABC) transporter, zinc finger protein, bZIP TF, serine/threonine kinase, sucrose nonfermenting (SNF) protein kinase, F-box protein, peroxidase, and polyketide synthase were specifically upregulated by Foc4 than by Foc1 during the degradation of the host cell wall. Furthermore, ∼26.6% of the Foc4-specific DEGs and 18.2% of the Foc1-specific DEGs induced by growth in the presence of host cell wall were unannotated. In addition, the number of specific DEGs related to pathogenicity in the comparison of P_Foc4 *vs.* G_Foc4 (142 DEGs) was slightly lower than for P_Foc1 *vs.* G_Foc1 (165 DEGs) (Table 3). However, more DEGs annotated as a G-protein, chitin synthase, ABC transporter, or bZIP TF were specifically upregulated by Foc4 than by Foc1 during the degradation of pectin. Additionally, ∼15.4% of the Foc4-specific DEGs and 14.0% of the Foc1-specific DEGs induced by growth with pectin were unannotated.

**Table 3:** The number of DEGs specifically regulated by Foc4 and Foc1 during decomposition of host cell wall polysaccharides, which were known to associated with fungal pathogenicity

Gene Function	BCW_Foc4vsG_Foc4		FCW_Foc1vsG_Foc1		P_Foc4vsG_Foc4		P_Foc1vsG_Foc1	
	Upregulation	Downregulation	Upregulation	Downregulation	Upregulation	Downregulation	Upregulation	Downregulation
Effector	1	2	0	0	0	1	0	0
SIX protein	0	3	0	1	0	2	0	0
G-protein	35	13	2	6	5	3	2	8
G-β	28	5	1	2	5	1	0	2
G-protein α subunit	2	2	0	0	0	1	0	1
G-protein β/γ-subunit complex binding	2	2	0	0	0	1	1	1
RagA G protein	5	3	0	0	0	1	1	1
G-protein-coupled receptor	4	6	0	4	0	0	0	5
Chitin synthase	1	1	0	0	0	0	0	0
Cutinase	0	2	0	3	0	1	0	3
Cutinase transcription factor	0	1	0	1	0	0	0	0
MFS transporter	18	25	35	20	16	12	9	21
ABC transporter	17	15	1	7	1	5	3	7
Zinc finger protein	46	34	14	19	4	25	9	12
Zn(II)_2_Cys_6_-type transcription factor	13	38	16	10	6	12	5	28
bZIP transcription factor	5	6	4	3	0	5	1	0
Serine/threonine kinase	23	21	7	9	1	8	5	4
Two-component system	1	0	2	0	0	0	1	0
Response regulator	2	1	2	2	0	1	2	1
Histidine kinase	2	4	2	2	1	1	3	0
MAPK/MAPKK/MAPKKK	0	4	0	1	0	0	1	0
SNF protein kinase	6	1	0	1	0	0	2	1
Other protein kinase	43	24	8	21	7	12	9	11
F-box protein	8	1	1	3	1	4	1	5
Peroxidase	1	0	0	0	0	0	0	0
Polyketide synthase	3	4	1	1	1	4	1	1
Cytochrome P450	2	13	7	4	0	3	1	8
No function	77	137	46	87	38	64	58	27

BCW, cell wall of Brazil cultivar; FCW, cell wall of Fengjiao cultivar; P, pectin; SIX, secreted in xylem; RagA, Ras-related GTP-binding protein A; MFS, major facilitator superfamily; ABC, ATP-binding cassette; bZIP, basic leucine zipper; MAPK, mitogen-activated protein kinase; SNF, sucrose nonfermenting.

Furthermore, 111 genes were specifically differentially expressed in BCW_Foc4 *vs.* G_Foc4 but not expressed in FCW_Foc1 *vs.* G_Foc1, including six DEGs that were upregulated and 105 DEGs that were downregulated (Table S16 in File S2). Additionally, 45 genes were specifically differentially expressed in P_Foc4 *vs.* G_Foc4 but not expressed in P_Foc1 *vs.* G_Foc1, including 9 DEGs that were upregulated and 36 DEGs that were downregulated (Table S17 in File S2). These genes may play essential roles as host-specific virulence genes when Foc4 invades Brazil banana cultivars. Among these DEGs, the genes known to be associated with fungal pathogenicity were all downregulated by Foc4 during the degradation of host cell wall polysaccharides (Table S18 in File S2). But the DEGs that were specifically upregulated in Foc4 grown in the presence of host cell wall or pectin were all genes of unknown function, which may need to be further characterized.

### DEGs encoding CWDEs induced by growth with host cell wall or pectin

The number of DEGs encoding putative CAZymes in each comparison and their expression profiles are shown in Figure S7 in File S1. Compared with Foc1, more DEGs encoding putative CAZymes were assigned to GHs, GTs, and CEs in Foc4 grown with host cell wall (Table S19 in File S2). During the degradation of host cell wall polysaccharides, the expression pattern of CAZyme genes assigned to each CAZy family in Foc4 was obviously different than in Foc1 (Figure S7G in File S1). Additionally, many genes encoding putative CAZymes were differentially expressed in a variety-specific manner when the two races degraded host cell wall polysaccharides. More specific DEGs encoding CAZyme were found in Foc4 than in Foc1 grown in the presence of host cell wall (Figure S7, A–F in File S1).

According to the analysis of DEGs encoding CWDE in Foc grown with host cell wall polysaccharides, the DEGs encoding hemicellulases and pectinases were the most abundant, followed by cellulases (Table S20 in File S2). Interestingly, the CWDE genes of Foc1 were more highly expressed during degradation of the host cell wall (Figure S8G in File S1). On the other hand, some genes involved in cellulose, hemicellulose, pectin, inulin, and starch degradation were specifically differentially expressed in the two races during the degradation of host cell wall polysaccharides (Figure S8, A–F in File S1). These results indicated that hemicellulases and pectinases produced by Foc might play a leading role in the degradation of the host cell wall, and that Foc1 may be better able to decompose banana cell wall than Foc4.

### DEGs related to pathogenicity induced by growth with host cell wall or pectin

**Figure 4:**
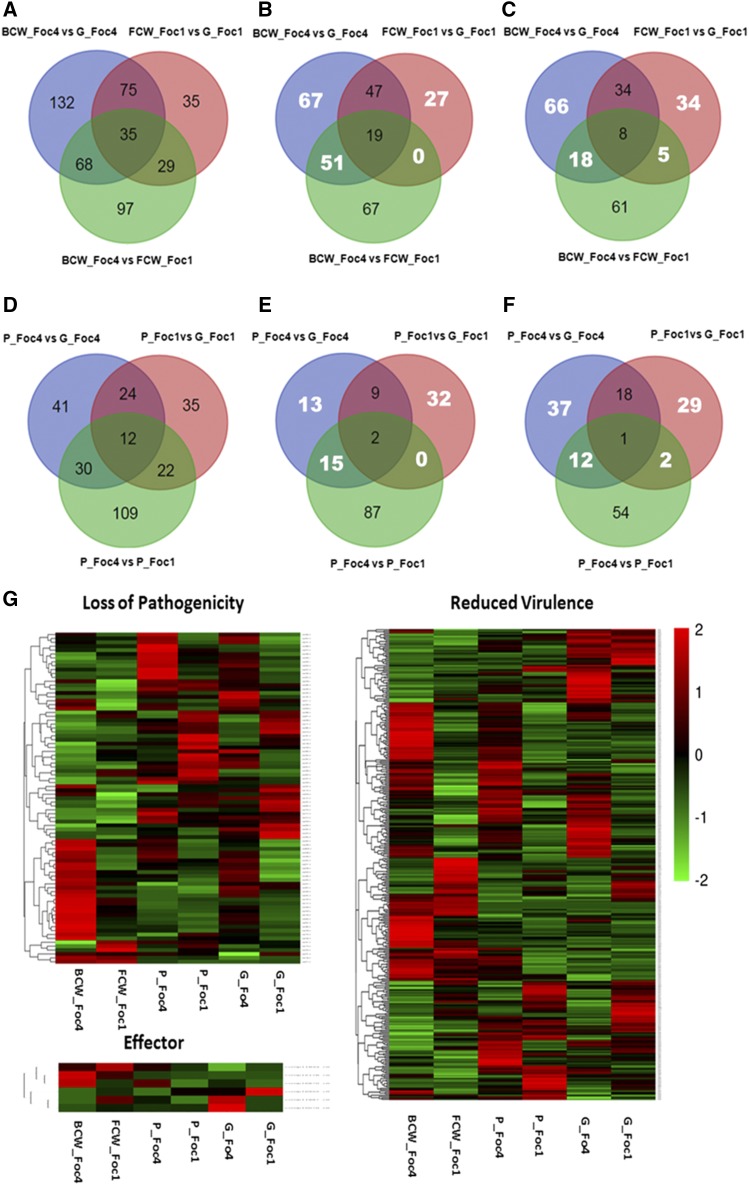
The expression profiles of genes related to host–pathogen interactions induced by host cell polysaccharides. The number of differentially expressed genes (DEGs) related to host–pathogen interactions showed in Venn diagram form (A–F). The number of DEGs (A), including upregulated (B) and downregulated DEGs (C), derived from comparisons induced by host cell wall. The number of DEGs (D), including upregulated (E) and downregulated DEGs (F), derived from comparisons induced by pectin. The numbers of specific DEGs homologous to pathogen–host interaction (PHI) genes regulated by Foc1 or Foc4 were shown in the nonoverlapping regions (white numbers). Expression profiles of putative pathogenicity genes homologous to genes associated with loss of pathogenicity, reduction of virulence, and pathogenic effectors in the PHI database were present in Heatmaps (G). Heatmaps represent fragments per kilobase of transcript per million fragments sequenced (FPKM) values of a unigene in each library.

The expression profiles of genes related to host–pathogen interactions in various pathogenic fungi and the number of these DEGs identified in each comparison are summarized in Figure 4. Overall, Foc4 expressed more PHI genes than Foc1 when degrading the host cell wall. The gene expression levels of these DEGs were higher in Foc4 than in Foc1 (Figure 4G). In addition, many more DEGs homologous to PHI genes were specifically regulated in Foc4 than in Foc1 when grown in the presence of host cell wall or pectin (Figure 4, A–F).

The genes associated with loss of pathogenicity and reduced virulence were more highly expressed in Foc4 than in Foc1 grown in the presence of host cell wall or pectin (Figure 4G). Furthermore, more DEGs associated with loss of pathogenicity and reduced virulence were specifically differentially expressed in Foc4 than in Foc1 grown in the presence of host cell wall (Figures S9 and S10 in File S1). Among the pathogenicity genes associated with loss of pathogenicity, DEGs encoding F-box (PHI: 490), signaling cascade (PHI: 182), and adenosine triphosphate (ATP) citrate lyase (PHI: 2386) proteins were specifically upregulated in Foc4 grown in the presence of host cell wall, while DEGs related to AF-toxin biosynthesis (PHI: 508), malate synthase (PHI: 2267 and PHI: 365,) and glyoxal oxidase (PHI: 352) were specifically upregulated in Foc1 grown in the presence of host cell wall. On the other hand, among the pathogenicity genes associated with loss of pathogenicity, DEGs encoding ATP citrate lyase (PHI: 2386) were specifically upregulated in Foc4 grown with pectin, while DEGs encoding phospholipase C (PHI: 2106), transcription activator (PHI: 481), and trehalose-6-phosphate synthase (PHI: 322, PHI: 2172, and PHI: 1064) were specifically upregulated in Foc1. Among the pathogenicity genes associated with reduced virulence, genes encoding pectin methylesterase (PME) (PHI: 1028 and PHI: 278), small secreted protein (PHI: 860), and apoptosis (PHI: 2334) proteins were found only among the DEGs that were specifically upregulated in Foc4 grown with host cell wall, while DEGs encoding cytochrome C peroxidase precursor (PHI: 854), hydrophobin (PHI: 458 and PHI: 487), and δ-aminolevulinic acid synthase (PHI: 2248) proteins were specifically upregulated in Foc1. In addition, more TFs orthologous to PHI genes involved in reduced virulence were differentially expressed in Foc4 than in Foc1 when grown in the presence of host cell wall. Of the pathogenicity genes involved in reduced virulence, DEGs related to alcohol oxidase (PHI: 199) and Ca^2+^ pump (PHI: 2095) proteins were specifically upregulated in Foc4 grown with pectin, while DEGs encoding phospholipase C (PHI: 2106) and a protein kinase (PHI: 1190) were specifically upregulated in Foc1 (Tables S8 and S21 in File S2). These results revealed that different pathogenicity genes are activated when two races decompose their particular host’s cell wall, and Foc4 exhibits a broader expression profile of pathogenicity genes than Foc1, which may also account for the variation in their virulence.

## Discussion

### Pectinases and hemicellulases may contribute greatly to the ability of Foc to decompose the host cell wall

A critical phase of pathogen infection in plants is penetration of the plant cell wall. Depending on the distinct host preference, pathogenic fungi require different CAZymes to decompose the plant cell wall. Genome analyses show that the content and distribution of CAZymes differ among pathogenic fungi. The genomes of *Botrytis cinerea* and *Sclerotinia sclerotiorum*, which tend to infect flowers and fruits rich in pectin, contain a larger number of CAZymes involved in pectin and hemicellulose decomposition than those involved in the decomposition of cellulose (Amselem *et al.* 2011). In contrast, the genomes of *Phaeosphaeria nodorum* and *Pyrenophora teres* f. teres, and *Magnaporthe oryzae*, whose hosts cell walls contain less pectin, exhibit higher proportions of CAZymes involved in cellulose and hemicellulose decomposition than of those involved in pectin decomposition (Amselem *et al.* 2011). Although the genome of Foc has been published, CWDEs remain uncharacterized in Foc (Guo *et al.* 2014). Analysis of the Foc transcriptome during early Cavendish variety infection showed that the two examined Foc isolates possess different CAZymes for attacking Brazil banana, and PL genes were mainly induced in Foc after infection for 48 hr (Guo *et al.* 2014). In this study, we annotated 393 CAZymes associated with plant cell wall degradation in the Foc transcriptome, among which a large proportion are involved in pectin and hemicellulose decomposition. Furthermore, we focused on the gene expression profiles of CWDEs during the degradation of host cell wall polysaccharides, which showed that Foc genes encoding pectinase and hemicellulase were strongly induced by the presence of host cell wall and pectin (Table S20 in File S2). Thus, our results suggest that the expression of genes encoding CWDEs in Foc during infection could be properly stimulated under *in vitro* growth conditions in the presence of host cell wall. In previous studies, the activity of polygalacturonases (PGs) and pectate lyases has been detected in the culture supernatant when Foc is grown in media containing pectin, which is also in agreement with our results (Dong and Wang 2010, 2015; Dong *et al.* 2011).

Although there was no obvious difference in the quantity of CWDE DEGs induced by host cell wall polysaccharides, the significantly different expression patterns of CWDE genes in the two races showed that Foc1 may be better able to decompose the host cell wall than Foc4 (Figure S8 in File S1). Plants can not only recognize cell wall-derived molecules following triggering of the plant defense response but also produce PG-inhibiting proteins to inhibit PG secreted by pathogens (Prade *et al.* 1999; Maulik *et al.* 2012; Zhang *et al.* 2014; Benedetti *et al.* 2015). The lower expression levels of CWDE genes in Foc4 are presumably associated with avoidance of the plant immune response. However, compared with Foc1 grown in the presence of host cell wall polysaccharides, a larger number of CAZymes genes were specifically regulated by Foc4, indicating that Foc4 exhibits a greater adaptive ability for different host nutrients.

Additionally, CWDEs are very important determinants of fungal pathogenicity. The roles of pectinases and hemicellulases in virulence have been investigated in several phytopathogens (Ten *et al.* 1998; Garcia-Maceira *et al.* 2000; Kars *et al.* 2005; Brito *et al.* 2006). In the present study, a DEG encoding PME orthologous to *Bcpme1* (PHI: 278) was specifically upregulated by Foc4 during degradation of the host cell wall. Disruption of *Bcpme1* in a *B. cinerea* strain led to a reduction of virulence in apple, grapevine, and *Arabidopsis thaliana* (Valettecollet *et al.* 2003).

### Genes involved in signaling may play important roles in Foc4 virulence

Protein kinases are responsible for the reversible phosphorylation of proteins, which play a major part in the regulation of fungal growth, developmental processes, and responses to environmental stimuli (Cohen 2000). The deletion of protein kinase genes results in loss of pathogenicity or low virulence of pathogenic fungi, as observed for the SNF protein kinase gene *FgSNF1* and the protein kinase gene *FgSsn3* in *F. graminearum*, as well as three MAPK genes (*FoSlt2*, *FoMkk2*, and *FoBck*) in Foc4 (Yu *et al.* 2014; Ding *et al.* 2015; Cao *et al.* 2016). In this study, GO molecular function analysis revealed that a large number of putative pathogenic factors that exhibit kinase activity were induced by growth in the presence of host cell wall polysaccharides. This result suggests that protein kinases may affect the transcription levels of CWDEs (Figure S3 in File S1). Mutation of the MAPK gene *fmk1* in Fol resulted in loss of pathogenicity in tomato plants and a serious reduction in the expression levels of *pl1*, encoding PL (Di Pietro *et al.* 2001). Additionally, more DEGs encoding protein kinases, including serine/threonine kinases and SNF kinases, were upregulated specifically in Foc4 than in Foc1 grown in the presence of host cell wall. Two DEGs that were specifically upregulated in Foc4 grown with host cell wall were orthologous to the Gin4-like protein kinase gene (PHI: 1184) and the Dbf2/Dbf20 protein kinase gene (PHI: 1187). Mutants with deletions of these genes showed a reduction of pathogenicity in *F. graminearum* (Wang *et al.* 2011).

Among signal transduction pathways, fungal TFs can regulate diverse biological processes. Zinc finger, bZIP, and Zn(II)_2_CyS_6_ TFs are considered to be pathogenicity-associated genes in some pathogenic fungi (Imazaki *et al.* 2007; Heimel *et al.* 2010; Chung *et al.* 2013; Qi *et al.* 2013; Shantappa *et al.* 2013; Yue *et al.* 2015). In this study, a set of TFs related to fungal pathogenicity was found to be activated during the degradation of host cell wall polysaccharides (Figure S3 in File S1). Additionally, more DEGs encoding zinc finger and bZIP TFs were upregulated specifically in Foc4 than in Foc1 grown with host cell wall. Nine DEGs specifically upregulated by Foc4 grown with host cell wall were similar to TFs (PHI: 1440, PHI: 1606, PHI: 1354, PHI: 1325, PHI: 1529, PHI: 1422, PHI: 1933, PHI: 1915, and PHI: 1556), including bZIP and zinc finger TFs. Mutations in these transcription factors result in multiple defects in virulence, growth, and toxin production in *F. graminearum* (Son *et al.* 2011).

Heterotrimeric GTP-binding proteins (G-proteins), consisting of Gα, Gβ, and Gγ subunits, are essential signaling components that mediate various cellular responses to environmental stimuli in eukaryotic organisms (Simon and Gautam 1991). In plant pathogenic fungi, G-proteins are indispensable for growth, asexual and sexual development, and virulence (Li *et al.* 2006). Mutations in G-protein genes generated in some fungi, such as *Cryphonectria parasitica*, *M. grisea*, *B. cinerea*, and *F. oxysporum* f. sp. *cucumerinum*, result in loss or reduction of pathogenicity (Choi *et al.* 1995; Liu and Dean 1997; Gronover *et al.* 2001; Jain *et al.* 2002). Compared with Foc1, Foc4 may activate FGA1-mediated G protein signaling during early Cavendish variety infection (Guo *et al.* 2014). In this study, DEGs including two G-protein α subunits, two G-protein β subunits, and four G-protein-coupled receptors were specifically upregulated in Foc4, while none of these genes were specifically regulated in Foc1 (Table 3). These results imply that some Foc4-specific G-protein α and β subunit-mediated signaling pathways may be activated during the degradation of the host cell wall compared with what occurs in Foc1.

In addition, F-box proteins, which are considered fungal pathogenic factors, mediate the ubiquitination of proteins targeted for degradation by the proteasome and play roles in signal transduction and regulation of diverse cell functions (Jonkers and Rep 2009; Jonkers *et al.* 2011; Guo *et al.* 2015). In this study, one DEG that was specifically upregulated in Foc4 grown with host cell wall was similar to *Frp1* (PHI: 490), which encodes an F-box protein, and a mutation in this gene in Fol results in loss of pathogenicity in tomato (Duyvesteijn *et al.* 2005).

Thus, more genes involved in signaling are specifically expressed in Foc4 than in Foc1, suggesting that Foc4 may be better able to adapt to host environments.

### Genes involved in fungal nutrition and transportation may play important roles in Foc4 virulence

The most obvious explanation for the interaction of fungi with host plants is to achieve more efficient nutrient acquisition, and fatty acids are one of the most basic nutritional elements. Mutations in peroxisome genes in the glyoxylate pathway, which is involved in fatty acid oxidation, lead to a loss of pathogenicity (Kimura *et al.* 2001). Peroxidase also protects pathogenic fungi against the oxidative stress generated by the host plant during infection (Tanabe *et al.* 2010; Singh *et al.* 2012). In our study, a DEG encoding peroxidase was upregulated specifically in Foc4 grown with host cell wall, while none of these genes were affected in Foc1 (Table 4).

Phytopathogenic fungi are champions of transmembrane transporters, which mediate the selective uptake and efflux of nutrients, metabolites, toxic compounds, and ions from their host environments. Therefore, transporters play a role in the acquisition of nutrients, pathogenesis, and fungicide sensitivity and resistance (Diallinas 2016). The ABC superfamily plays a role in protecting pathogenic fungi against plant defense compounds at an advanced phase of disease, which has been characterized in various phytopathogenic fungi (Stergiopoulos *et al.* 2002, 2003; Sun *et al.* 2006; Gupta and Chattoo 2008). The major facilitator superfamily (MFS) transporters are largely known as secondary active carriers that play an important role in antifungal drug resistance and pathogen–host interactions (Costa *et al.* 2014). In our study, more DEGs for ABC transporters were upregulated specifically in Foc4 than in Foc1 grown with host cell wall (Table 4). When grown with host cell wall, one of the DEGs that was specifically upregulated in Foc4 was orthologous to the MFS transporter-like gene *CTB4* (PHI: 737 and PHI: 2329). A *Cercospora nicotianae CTB4* mutant displays a drastic reduction of cercosporin toxin accumulation and fungal virulence (Choquer *et al.* 2007).

During degradation of the host cell wall, genes involved in fungal nutrition and transportation may be required for Foc4 virulence against plant defense.

### Effectors, chitin synthase, and polyketide synthase may play a role in Foc4 infection

Effectors are small molecular weight secreted proteins produced by pathogenic fungi at various stages of infection that can alter host cell metabolism, inhibit or stimulate effector-triggered immune responses, and facilitate infection (Ellis *et al.* 2009; Wit *et al.* 2009). Chitin synthases contribute to the structural integrity of the fungal cell wall and are important for fungal growth, development, and pathogenicity (Werner *et al.* 2007). Polyketide synthases, which are involved in the biosynthesis of fungal toxins, have been shown to participate in the regulation of fungal virulence (Takano *et al.* 1995; Tsai *et al.* 1998; Bohnert *et al.* 2004). In our study, slightly more DEGs encoding these pathogenic factors were specifically upregulated in Foc4 than in Foc1 grown with host cell wall (Table 4). Under induction by host cell wall, one of the DEGs that was specifically upregulated by Foc4 was orthologous to *MSP1* (PHI: 860), which encodes a small secreted protein required for the virulence of *M. grisea* (Jeong *et al.* 2007). Another DEG that was specifically upregulated in Foc4 is orthologous to *CgCHSIII* (PHI: 1055), encoding chitin synthase, which is expressed during pathogenic development but does not affect pathogenicity of *Colletotrichum graminicola* (Werner *et al.* 2007). Overall, these genes may be activated by growth in the presence of host cell wall and may also play important roles in Foc4 virulence at different stages of infection.

### Conclusions

A comprehensive transcriptome resource for Foc was generated in this study, and the gene expression profiles of fungi grown in the presence of host cell wall polysaccharides were characterized using RNA-seq. Comparative transcriptome analysis allowed the identification of a set of DEGs when Foc decomposed host cell wall polysaccharides, revealing the molecular mechanisms of pathogenesis in banana and the genetic basis of host specificity in Foc. In this study, a large number of Foc4- and Foc1-specific regulated genes induced when fungi grew with host cell wall polysaccharide were first characterized, including unknown genes. In particular, the results indicated that genes that are specifically regulated by Foc4, especially genes involved in penetration of the host cell wall, signaling, and transportation, may have a significant impact on virulence. Other pathogenicity factors also attracted our attention. Functional analysis of these Foc genes needs to be carried out to discover new pathogenicity-related or virulence genes.

## Supplementary Material

Supplemental material is available online at www.g3journal.org/lookup/suppl/doi:10.1534/g3.117.042226/-/DC1.
