# Supplementary material for: Comparative Transcriptomic Analysis of Race 1 and Race 4 of Fusarium oxysporum f. sp. cubense Induced with Different Carbon Sources
Source: G3 (Bethesda). 2017 May 3;7(7):2125–38. doi: 10.1534/g3.117.042226 (PMC5499122; doi:10.1534/g3.117.042226)
Supplement: Supplementary file 1 [file 2125FileS1.docx]

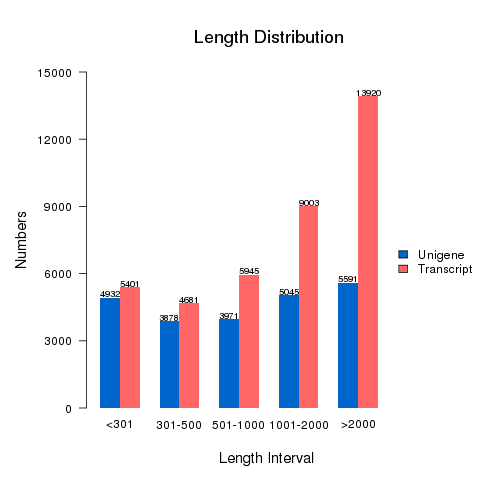


**Figure S1 Frequency distribution of six Foc sample.**


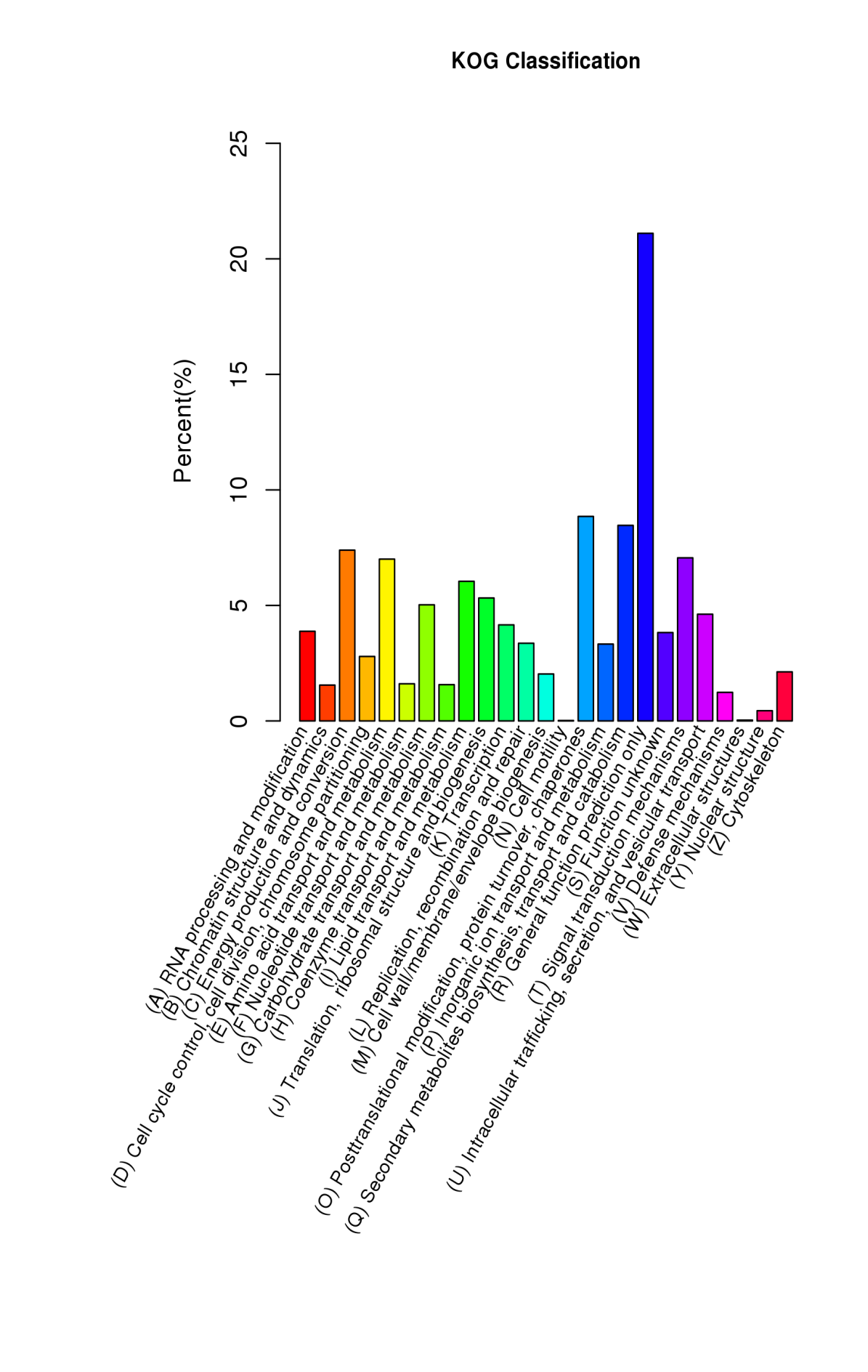


F**igure S2 KOG functional classification of all unigenes sequences.** Unigenes aligned to the KOG database can be classified functionally into 25 molecular families.


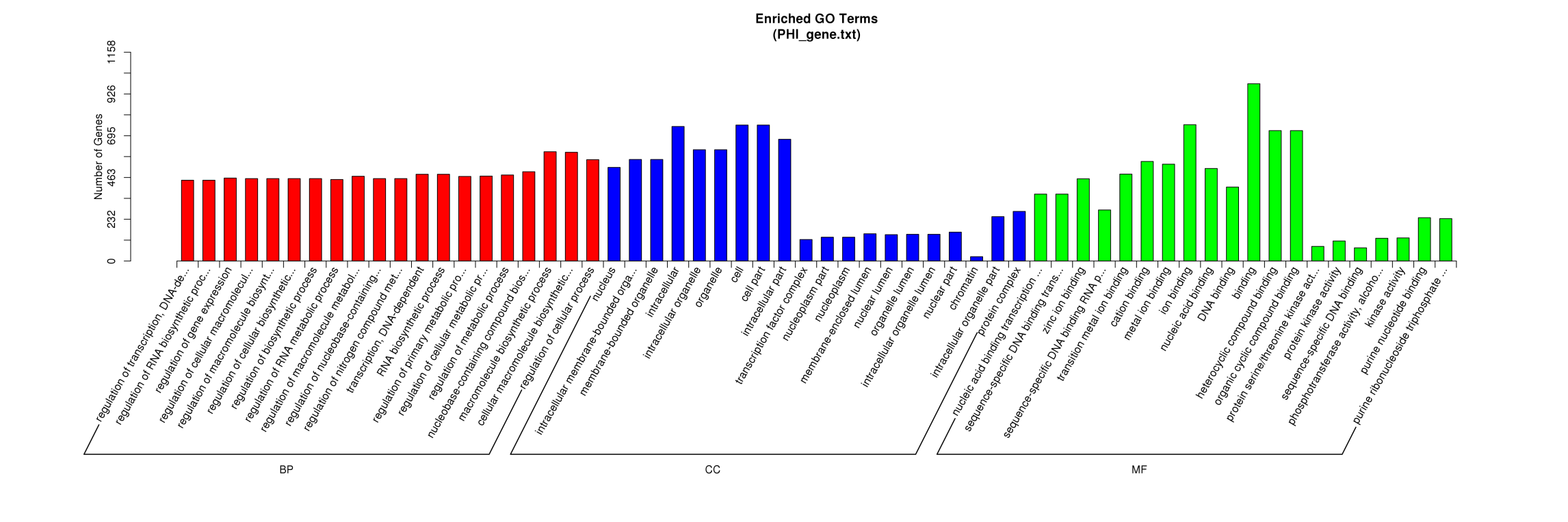


**Figure S3** **GO term assignments to Foc unigenes with homology to PHI genes.** The results are summarized in three main categories, Biological process, Cellular component, and Molecular function. The right y-axis indicates the number of genes in a category. The left y-axis indicates the percentage of a specific category of genes in that main category.


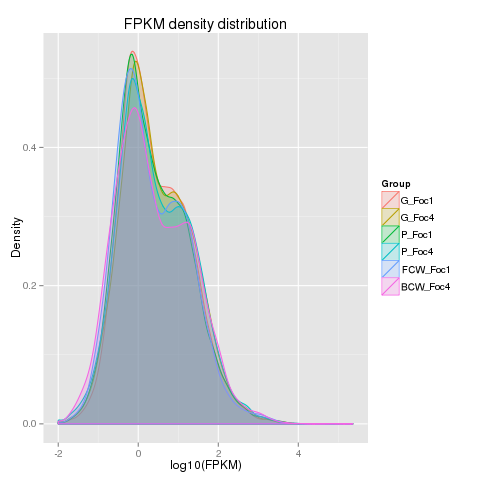


**Figure S4 Frequency distribution of six Foc sample by expected by fragments per kilobase of transcript per million fragments sequenced (FPKM).**


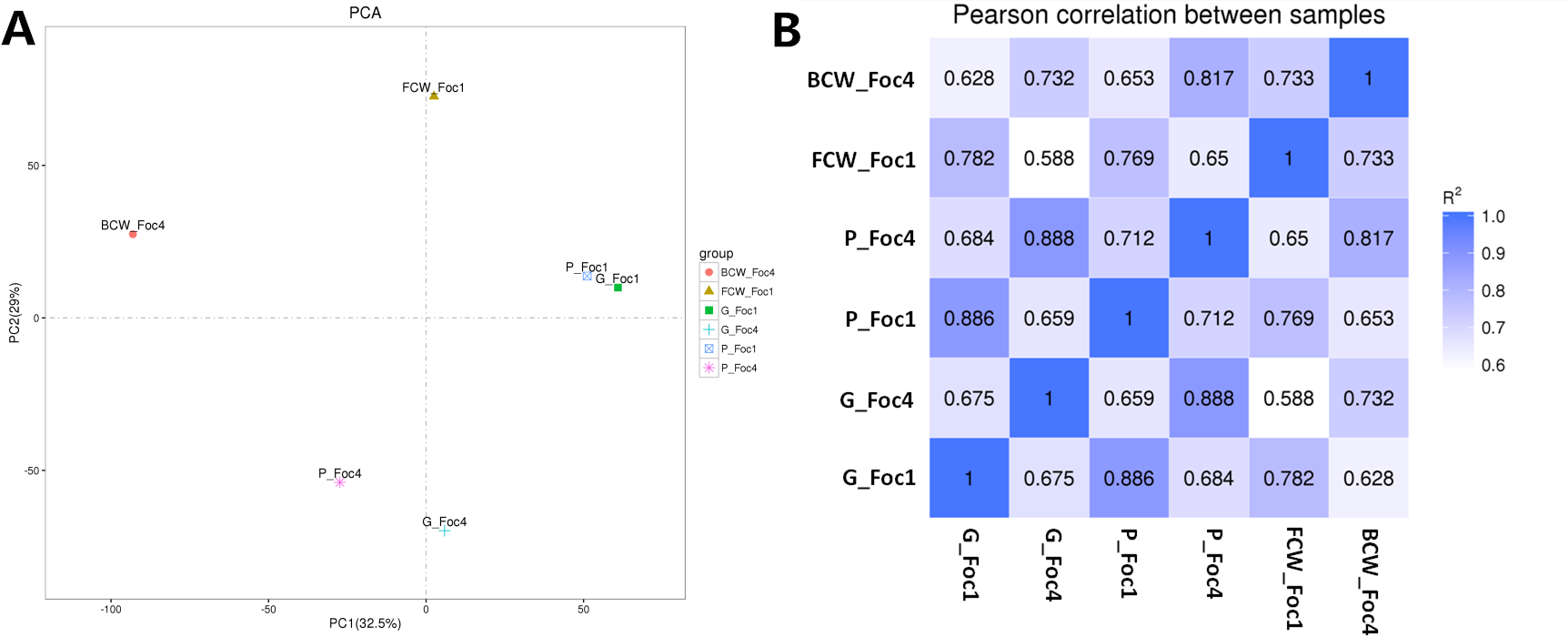


**Figure S5 Multivariate grouping of experimental libraries based on the expression profiles of genes from six Foc libraries using PCA (A). The Pearson correlation between** **six Foc libraries (B).**


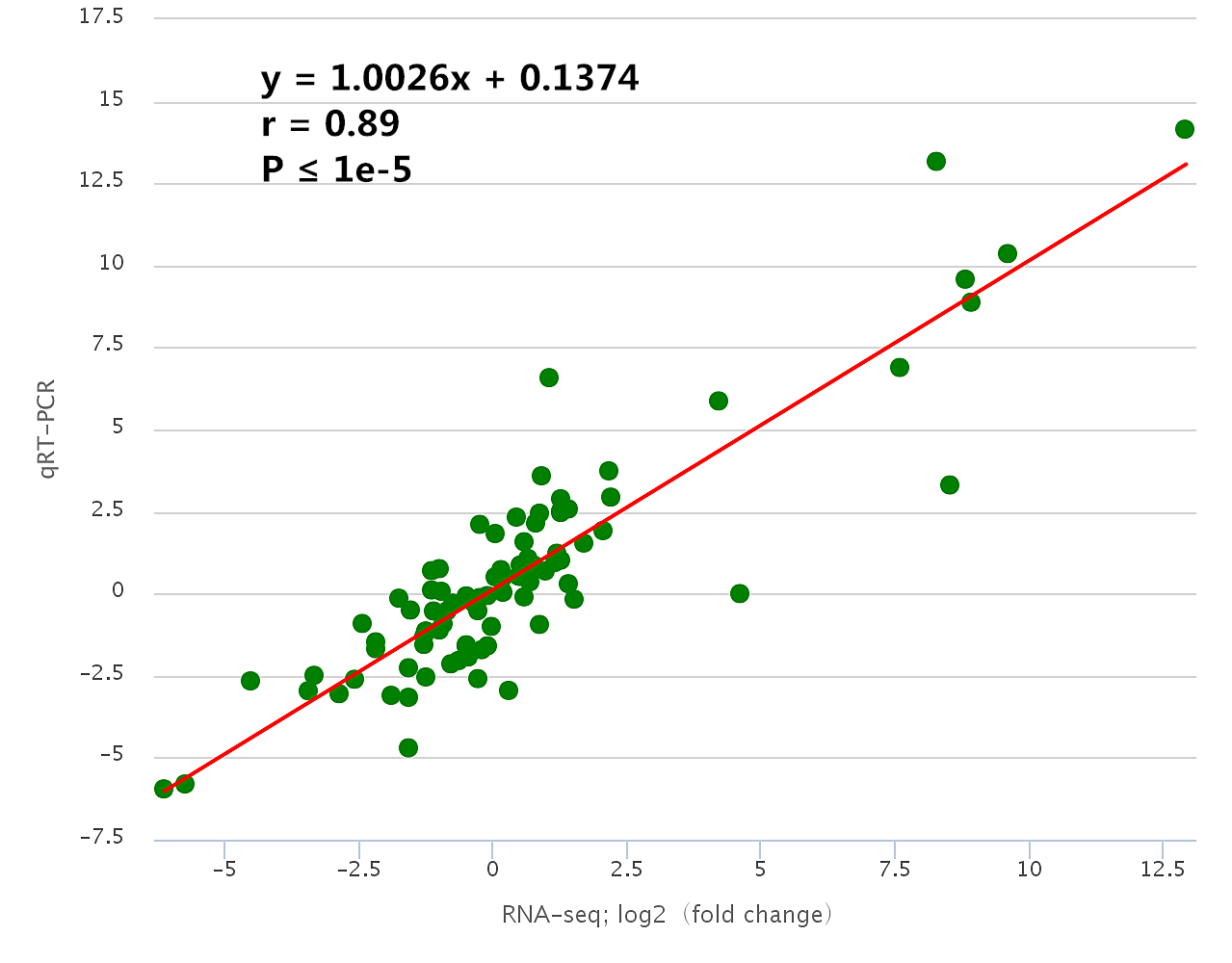


**Figure S6 Correlation analysis of 15 randomly selected differentially expressed unigenes induced by host cell wall polysaccharides base on RNA-seq and qRT-PCR data.**


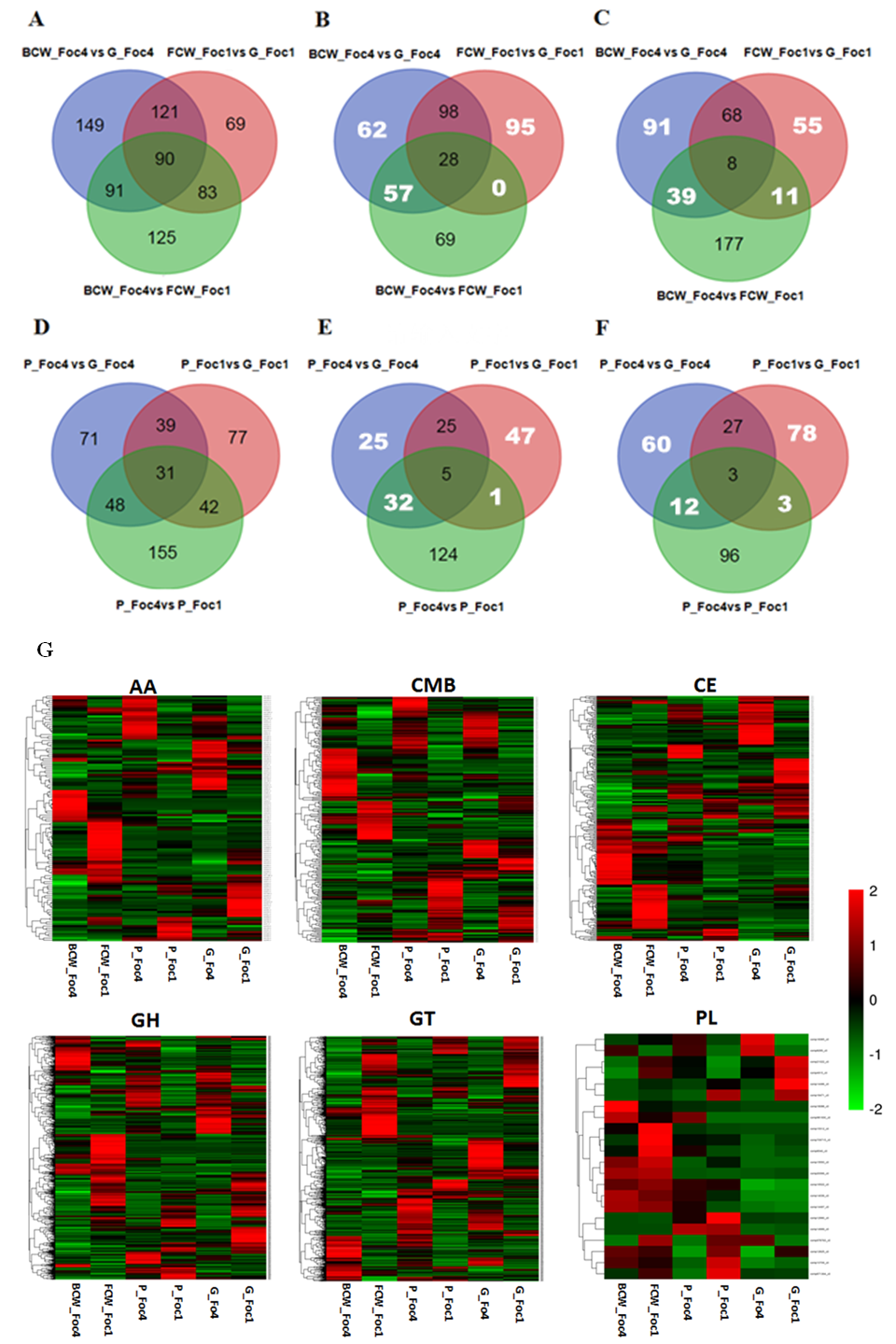


**Figure S7 The expression profiles of genes encoding carbohydrate-active enzymes induced by host cell wall polysaccharides.** The number of DEGs (A), including up-regulated (B) and down-regulated DEGs (C), derived from comparing cells grown in the presence of host cell wall. The number of DEGs (D), including up-regulated (E) and down-regulated DEGs (F), derived from comparing cells grown in the presence of pectin. The numbers of specific DEGs encoding CAZymes, regulated by Foc1 or Foc4, were shown in the non-overlapping regions (white numbers). Expression profiles of CAZymes genes sorted according to the type of CAZyme modules were present in Heatmaps (G). Heatmaps represent FPKM values of a unigene in each library. CAZymes Family: CBM, carbohydrate-binding modules; GH=glycoside hydrolases; GT, glycosyl transferases; PL, polysaccharide lyases; PME, pectin methyl esterase; CE, carbohydrate esterases.


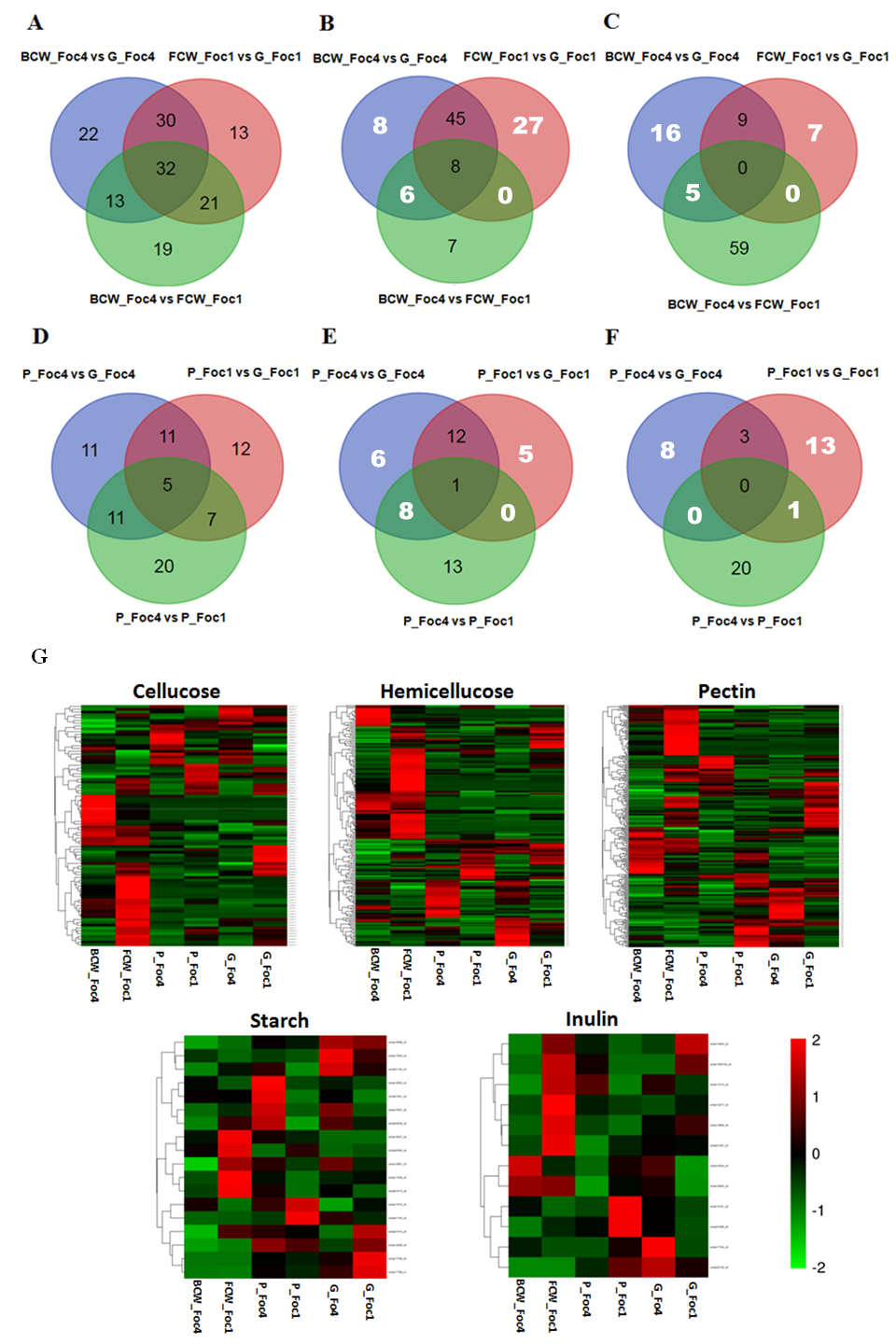


**Figure S8 The expression profiles of genes encoding cell wall degrading enzyme induced by host cell polysaccharides.**The number of specific and common DEGs encoding CWDE showed in Venn diagram form (A-F). The number of DEGs (A), including up-regulated (B) and down-regulated DEGs (C), derived from comparisons induced by host cell wall. The number of DEGs (D), including up-regulated (E) and down-regulated DEGs (F), derived from comparisons induced by pectin. The numbers of specific DEGs encoding CWDEs, regulated by Foc1 or Foc4, were shown in the non-overlapping regions (white numbers). Expression profiles of CWDE genes involved in different plant polysaccharide degradation were present in Heatmaps (G). Heatmaps represent FPKM values of a unigene in each library.


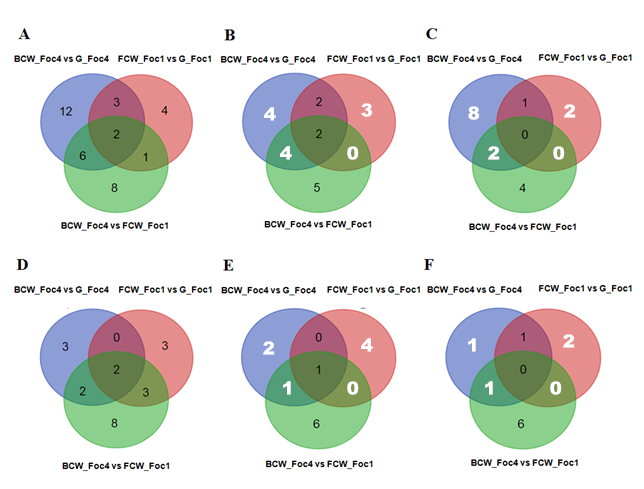


**Figure S9 Number of specific and common DGEs** **orthologous to PHI genes associated with loss of pathogenicity in cells grown with host cell wall polysaccharides.** The number of DEGs (A), including up-regulated (B) and down-regulated DEGs (C), derived from comparing cells grown in the presence of host cell wall. The number of DEGs (D), including up-regulated (E) and down-regulated DEGs (F), derived from comparing cells grown in the presence of pectin. The number of specific DEGs identified in different comparisons, located in non-overlapping regions, respectively.


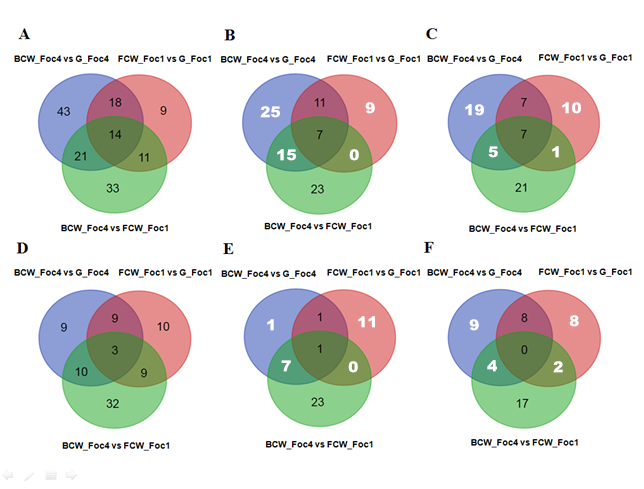


**Figure S10 Number of specific and common DGEs orthologous to PHI genes associated with reduced virulence in cells grown with host cell wall polysaccharides.** The number of DEGs (A), including up-regulated (B) and down-regulated DEGs (C), derived from comparing cells grown in the presence of host cell wall. The number of DEGs (D), including up-regulated (E) and down-regulated DEGs (F), derived from comparing cells grown in the presence of pectin. The number of specific DEGs identified in different comparisons, located in non-overlapping regions, respectively.
